# Biophysics of Malarial Parasite Exit from Infected Erythrocytes

**DOI:** 10.1371/journal.pone.0020869

**Published:** 2011-06-17

**Authors:** Rajesh Chandramohanadas, YongKeun Park, Lena Lui, Ang Li, David Quinn, Kingsley Liew, Monica Diez-Silva, Yongjin Sung, Ming Dao, Chwee Teck Lim, Peter Rainer Preiser, Subra Suresh

**Affiliations:** 1 Singapore-MIT Alliance for Research and Technology Centre, Singapore, Singapore; 2 George R. Spectroscopy Laboratory, Massachusetts Institute of Technology, Cambridge, Massachusetts, United States of America; 3 Department of Materials Science and Engineering, Massachusetts Institute of Technology, Cambridge, Massachusetts, United States of America; 4 National University of Singapore, Singapore, Singapore; 5 Nanyang Technological University, Singapore, Singapore; University of Bern, Switzerland

## Abstract

Upon infection and development within human erythrocytes, *P. falciparum* induces alterations to the infected RBC morphology and bio-mechanical properties to eventually rupture the host cells through parasitic and host derived proteases of cysteine and serine families. We used previously reported broad-spectrum inhibitors (E64d, EGTA-AM and chymostatin) to inhibit these proteases and impede rupture to analyze mechanical signatures associated with parasite escape. Treatment of late-stage iRBCs with E64d and EGTA-AM prevented rupture, resulted in no major RBC cytoskeletal reconfiguration but altered schizont morphology followed by dramatic re-distribution of three-dimensional refractive index (3D-RI) within the iRBC. These phenotypes demonstrated several-fold increased iRBC membrane flickering. In contrast, chymostatin treatment showed no 3D-RI changes and caused elevated fluctuations solely within the parasitophorous vacuole. We show that E64d and EGTA-AM supported PV breakdown and the resulting elevated fluctuations followed non-Gaussian pattern that resulted from direct merozoite impingement against the iRBC membrane. Optical trapping experiments highlighted reduced deformability of the iRBC membranes upon rupture-arrest, more specifically in the treatments that facilitated PV breakdown. Taken together, our experiments provide novel mechanistic interpretations on the role of parasitophorous vacuole in maintaining the spherical schizont morphology, the impact of PV breakdown on iRBC membrane fluctuations leading to eventual parasite escape and the evolution of membrane stiffness properties of host cells in which merozoites were irreversibly trapped, recourse to protease inhibitors. These findings provide a comprehensive, previously unavailable, body of information on the combined effects of biochemical and biophysical factors on parasite egress from iRBCs.

## Introduction

The human malarial parasite, *Plasmodium* (*P.*) *falciparum* invades and develops within host red blood cells (RBCs) during the 48-hour asexual cycle. During this period, parasites undergo progression from single-nucleated rings to multi-nucleated schizonts by consuming nutrients generated by degrading hemoglobin within a digestive vacuole (DV). The progenies formed known as merozoites then break open the membrane and cytoskeleton of the infected RBCs (iRBCs) to establish a new cycle of infection. As the parasites mature and differentiate within a growth-permissive parasitophorous vacuole (PV), the iRBCs undergo extensive morphological alterations [1], [2]. Towards the end of parasite's intra-erythrocytic life cycle, the initially biconcave iRBCs become more spherical in shape and less deformable. Nanoscale knobs formed on their external surfaces facilitate significantly increased cytoadherence to the endothelium of the vasculature to ensure diminished clearance in spleen [3]. Increased rigidity and adherence are linked to malaria pathology and are attributed to the export of several parasite proteins such as RESA [4], PfEMPs [5] and KHARP [6] that bind to cytoskeleton and modify the biophysical properties of the iRBC membrane.

Proteases orchestrate major pathways during the intra-erythrocytic stages of parasite development. In total, the *P. falciparum* genome codes about 100 proteases that are actively transcribed [7], the major function being hemoglobin degradation within the food vacuole. Other roles of malarial proteases include merozoite invasion into host cells [8], [9], priming of proteins for export into their respective compartments [10] as well as rupturing of the iRBCs to complete an erythrocytic cycle. Several parasite proteases are known to be effectors of merozoite egress through tightly regulated proteolytic activation cascades such as serine repeat antigens, dipeptidylaminopeptidase - 3 and subtilisin-1 like protease [11], [12], [13]. Recently, host-derived calpains were shown to be responsible for cytoskeletal degradation of the iRBC towards later stages of schizogony thereby enforcing rupture [14]. This is supported by the observation that global Ca^2+^ re-distribution from the food vacuole to the PV space occurs just prior to rupture [15]. Merozoite egress is an explosive event and likely involves sequential disruption of PV and iRBC membranes that could be differentially blocked with cysteine/ serine protease inhibitors of different specificities such as E64d, chymostatin and leupeptin [16].

When the merozoites are about to be released, the iRBC undergoes morphological and physiological changes such as formation of flower-shaped structures [17] enlargement of the parasitophorous vacuole [18], increased permeability of the iRBC membrane [19] and regulated activation of proteases to essentially destabilize the cytoskeleton to facilitate egress. However, how these connections between biophysical and biochemical changes lead to iRBC rupture remains largely unknown. Blocking rupture by recourse to protease inhibitors while the merozoites prepare to exit the host iRBCs thus provides an important path to investigate the mechanisms of egress. In this work, we have employed hitherto unexplored biochemical and biophysical approaches to evaluate the molecular events associated with merozoite egress from *P. falciparum*-infected RBCs. After treatment with irreversible cysteine protease inhibitors (e.g. E64d), mature & viable merozoites were locked within the iRBCs that had lost their characteristic spherical shape, but with no apparent cytoskeletal reconfiguration. In our experiments, we observed global re-distribution of 3D-RI within the un-ruptured iRBCs in E64d and EGTA-AM treatments indicating PV breakdown. Also, remarkably elevated membrane fluctuations (flickering) were observed in these treatments. We analyze and synthesize these observations to develop novel mechanistic interpretations of the role of PV in providing shape to a schizont-stage iRBC, the sequence of events leading to membrane breakdown during parasite escape and the evolution of membrane stiffness properties of iRBCs in which parasites are irreversibly locked.

## Results

### Protease inhibitors cause differential blockage of parasite egress

**Figure 1:**
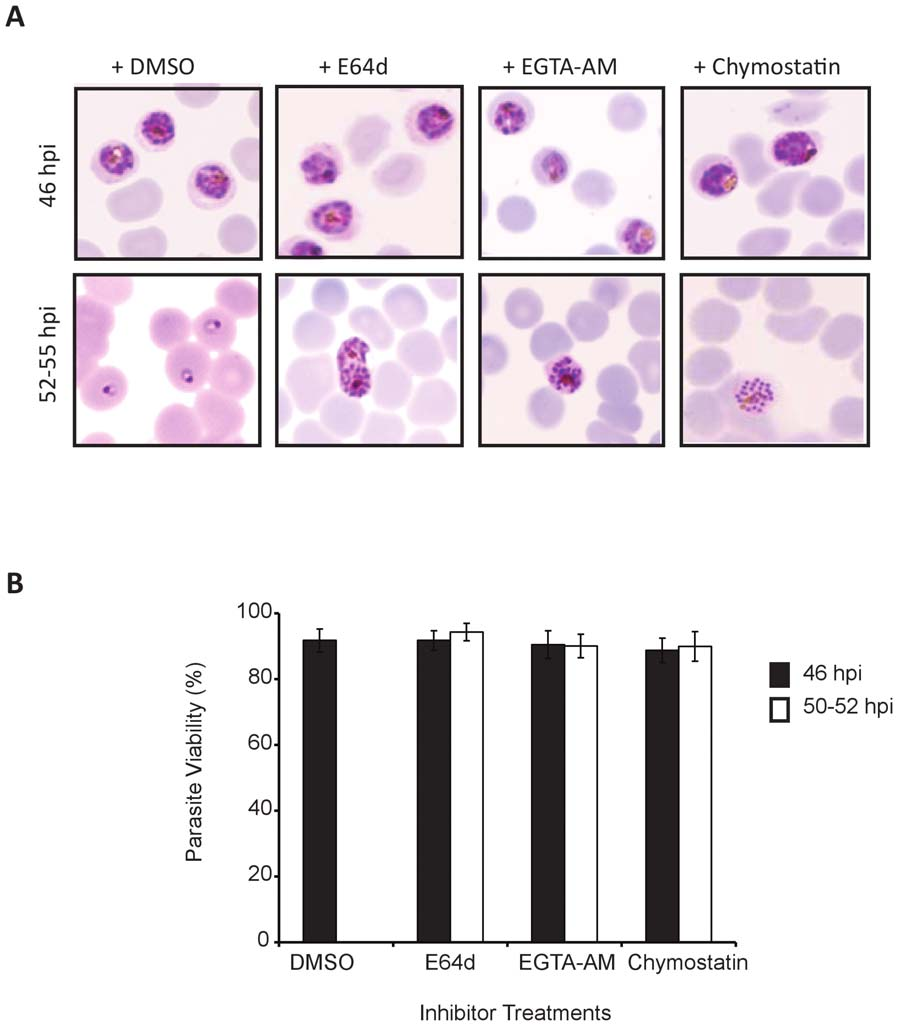
Protease inhibitors arrest merozoite egress. (**A**) Schizont-stage iRBCs (∼44 hpi) were treated with DMSO, E64d, EGTA-AM or Chymostatin and were followed by giemsa staining and microscopy. Morphology of iRBCs was analyzed at 46 hpi (Upper Panel) and 55 hpi (Lower Panel). While all schizont-stage iRBCs from DMSO-treated samples ruptured and established ring stage infections by 55^th^ hpi, merozoites from inhibitor-treated iRBCs failed to egress. (**B**) Viability of inhibitor-treated iRBCs was analyzed by JC-1 staining and flow cytometry. The values are expressed as percentage of healthy schizonts gated based on JC-1 and DAPI staining before and after inhibitor treatments; error bars represent standard deviation.

Schizont-stage *P. falciparum* infected RBCs, approximately 44 h post-invasion (hpi) were treated with protease inhibitors E64d, EGTA-AM and chymostatin that are known to block parasite egress. E64 or a more cell permeable version E64d primarily inactivates thiol proteases [14], [15], [16] and primarily host-calpain 1, EGTA-AM irreversibly chelates calcium ions required for calpain activation [14] while chymostatin can inactivate both cysteine and serine family proteases involved in rupture. In our experiments, all inhibitors irreversibly locked merozoites within the iRBCs at schizont stage while the DMSO-treated controls established new ring stage infections by 50–52 hpi (Fig. 1A). No food vacuole swelling effect was observed in the schizont-stage parasites treated with inhibitors. The rupture phenotypes resulted by E64d and EGTA-AM treatments appeared highly fragile structures from giemsa smears, and a fraction of them had undergone bursting when smeared. Merozoites from rupture-phenotypes appeared less invasive upon removal of inhibitors -compared to DMSO-treated iRBCs, possibly due to partial inhibition of invasion related proteases by the broad-spectrum inhibitors used in this study. However, the parasites that were locked inside the host cells were metabolically active as observed from vitality staining experiments (Fig. 1B). Minimal parasite death was observed in cultures treated with inhibitors, when iRBCs stained with JC-1 were used to monitor mitochondrial potential [20], [21], [22] as a measure of viability (Fig. S1A) before and actual rupture time points, while treatment with compounds such as chloroquine or CCCP caused death of a major fraction of parasites within 2 hours (Fig. S1B).

Using a parasite line expressing GFP in the parasite cytoplasm/food vacuole, we further analyzed the effect of inhibitor treatment on PV integrity using western blots. E64d blocked iRBC membrane rupture, but supported PV membrane degradation as confirmed by leakage of GFP into iRBC cytosol in rupture phenotypes as previously reported [23]. A similar effect was observed in rupture-phenotypes caused by EGTA-AM treatment. However, chymostatin appeared to block rupture of both PV and RBC membranes, with no apparent detectable leakage of GFP into RBC cytosol (Fig. S2). In order to examine the surface morphology and cytoskeletal structures of the iRBCs in which parasites were trapped, we used atomic force microscopy (AFM) as described elsewhere [24]. Schizont-stage iRBCs treated with inhibitors were compared to the corresponding DMSO-treated controls before and after the actual rupture time points. The external surface of rupture arrested iRBCs (50–52 hpi) was rougher compared to schizonts treated with similar dose of inhibitors, but harvested prior to the rupture time point (∼46 hpi) and those treated with DMSO (Fig. S3, right panel). This could be due to increased pressure continuously exerted by merozoites, osmotic factors or an altered hydration state of the membrane in rupture-arrested phenotypes. The high-resolution images obtained from AFM were processed to extract information on the integrity and morphology of the cytoskeletal network (Fig. S3, left panel). Typically as the parasite develops, progressive expansion of red cell cytoskeleton occurs leading to schizont stage iRBCs with less dense cytoskeletal structures as demonstrated by increased mesh size of the spectrin-based cytoskeleton. In our experiments, we observed cytoskeletal patterns that are characteristic of schizonts, which remained mostly comparable during treatments irrespectively of the inhibitors used and the duration of treatment. This implies that all the inhibitors presumably blocked any further damage and disintegration of the iRBC cytoskeletal network, thereby locking the merozoites within.

### E64d and EGTA-AM causes re-distribution of 3-Dimensional Refractive Index (3D-RI)

**Figure 2:**
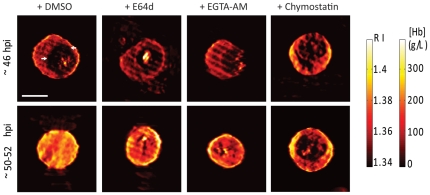
3D-RI distribution in inhibitors treated iRBCs. Schizont-stage iRBCs (∼44 hpi) treated with DMSO, E64d, EGTA-AM or Chymostatin and harvested at 46 hpi (Upper Panel) and 50–52 hpi (Lower Panel) were analyzed by tomographic phase microscopy (TPM). The images are lateral cross-sections of 3D- refractive index distributions at the focused plane. iRBCs at 46 hpi showed interior structures with low RI, corresponding to parasitophorous vacuole (Upper Panel-white arrow heads). However, inhibitor-treated iRBCs harvested around 52 hpi showed homogeneous distribution of 3D-RI with an apparent lack of visible PV. Scale bar, 5 µm. Color maps show the refractive index (Left) and the Hb concentration (Right). Figure shows representative images from 20 measured tomograms for each group.

Refractive index (RI) distributions within iRBCs are known to be quantitative and qualitative indicators of biochemical and morphological changes arising from parasitization [25]. 3D-RI distributions inside parasitized RBCs were measured using tomographic phase microscopy (TPM) as reported before [25], [26]. The physical principle of TPM is to record the optical electromagnetic fields of a sample at various angles of illumination and then retrieve 3D-RI distribution of the sample by solving the inverse light projection problem [26]. Typically, schizont-stage iRBCs show non-homogeneous distribution of RI, mainly due to the complex compartmentalization of parasites within the PV (with low refractive index) surrounded by RBC cytosol (with high RI). Schizont-stage iRBCs treated with inhibitors and harvested before rupture time (44–46 hpi) showed characteristic 3D-RI pattern (Fig. 2, row of images in upper panel), with an apparent PV (indicated by white arrow heads). However, rupture phenotypes (50–52 hpi) caused by E64d and EGTA-AM treatments suggested extensive re-distribution of RI (Fig. 2, row of images in lower panel). These iRBCs were relatively more homogeneous and were characterized by loss of the low RI region corresponding to the PV. Chymostatin-treated, rupture-impaired iRBCs were largely similar to early stage schizont with an intact PV and RBC membrane. 3D-RI information can be directly related to hemoglobin concentration, since the most abundant component in the erythrocyte cytoplasm is hemoglobin. Taken together, it demonstrates that E64d and EGTA treatments prevented the merozoites from escaping the host cell, but showed no influence on PV breakdown. However, merozoites were trapped within the iRBC membrane.

### E64d and EGTA treatment alter iRBC morphology and increase membrane fluctuations

**Figure 3:**
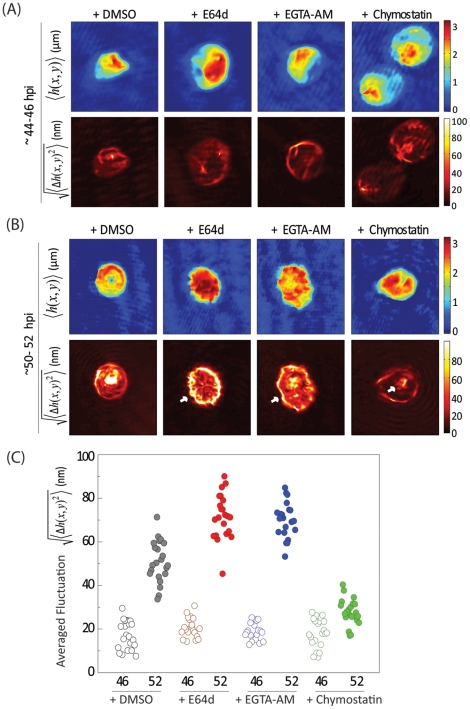
Cell thickness maps and membrane fluctuations from inhibitor-treated iRBCs. (**A**) Cell thickness maps (Upper Panel) and magnitude of membrane fluctuations (Lower Panel) of inhibitor treated iRBCs and corresponding DMSO-treated controls harvested at 46 hpi were generated using diffraction phase microscopy (DPM). (**B**) Morphology (Upper Panel) and magnitude of membrane fluctuations (Lower panel) from rupture-arrested iRBCs harvested around 50 hpi. Unlike the earlier time point, rupture-phenotypes from E64d and EGTA-treatments showed schizonts that had lost spherical shape and with elevated membrane fluctuations, especially around the edges. Chymostatin-treated, rupture-arrested iRBCs showed no increase in iRBC membrane fluctuations (**C**) Averaged membrane fluctuations were calculated over the entire area of iRBCs harvested from 46 hpi and 50 hpi. DMSO-treated controls showed increase in fluctuations (accounts for transition from schizont to ring) while E64d and EGTA-AM caused elevated membrane fluctuations in rupture phenotypes. Chymostatin treated samples showed no transition.

The cell membrane fluctuations or flickering demonstrates changes in cytoskeletal structure, membrane physical properties, as well as pathological state of the cell [25], [29]. We utilized diffraction phase microscopy (DPM) to quantify cell thickness [27], [28] with nm level sensitivity and ms temporal resolution. Dynamic membrane thickness maps were measured from iRBCs treated with inhibitors but harvested before the rupture time point (∼46 hpi) and corresponding rupture-arrested phenotypes (∼52 hpi) using DPM. Time-averaged cellular morphology, 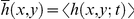 was calculated by averaging the consecutive cell thickness maps 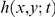 at time *t*. In order to quantitatively probe dynamic membrane fluctuations, we analyzed the membrane displacement information by subtracting the averaged shape from the cell thickness map, 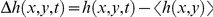. We then calculated the root-mean-squared (RMS) displacement of membrane fluctuations, 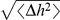, which covers the entire cell area for 2 s at 120 frames/s. Schizont stage iRBCs treated with inhibitors and harvested before the rupture time point (∼46 hpi) were similar to non-treated schizonts (Fig. 3A) with classical spherical morphology. However, our experiments demonstrated significantly different morphology of rupture-arrested phenotypes (50–52 hpi) due to E64d and EGTA-AM treatments, which appeared as flatter structures that had lost their characteristic schizont-stage spherical shape (Fig. 3B). Also closer analysis showed larger fluctuations in E64d and EGTA-treated rupture-arrested phenotypes, especially around the edges of iRBCs. DMSO-treated controls that had ruptured and invaded into fresh RBCs showed characteristic large fluctuations of ring stage iRBCs as previously reported [25]. In contrast, rupture-arrested phenotypes resulting from chymostatin treatment showed smaller fluctuations in the RBC membrane, but increased fluctuations within the PV compartment, suggesting primed merozoites that are unable to break open the PV.

It has been reported that during intra-erythrocytic development of *P. falciparum*, dynamic membrane fluctuations of the iRBCs gradually decrease, which indicates increased membrane stiffness [25]. From our measurements, we calculated averaged membrane fluctuations over the total cell area after deducting the fluctuations observed around the edges as well as that from the area of PV. The DMSO-treated controls showed a clear transition from schizonts (with relatively less membrane fluctuations) to ring-stage iRBCs (with higher levels of flickering) as expected. In the rupture-arrested iRBCs (50–52 hpi) due to E64d and EGTA-AM treatments, up to 3-fold enhancements in membrane fluctuations were observed as compared to schizonts harvested before rupture time point (46 hpi) from the corresponding treatments (Fig. 3C). Chymostatin-treated schizonts showed no significant changes in averaged fluctuations before and after rupture time points. Interestingly, E64d and EGTA-AM induced rupture-arrest resulted in elevated iRBC membrane fluctuations (60–80 nm) that were even higher in magnitude than typical ring-infected or un-infected RBCs (∼50 nm). Hence we analyzed the membrane fluctuation data in greater detail to further target the possible reasons for highly elevated membrane fluctuations upon rupture-arrest.

### Increased membrane fluctuations were most likely caused by the repeated impingement of merozoites on iRBC membrane

The membrane fluctuations of a normal healthy RBC are driven by two sources of energy: (i) thermal energy – the hitting of small molecules to the membrane, and (ii) metabolic energy- the phosphorylation by ATP, that can remodel the membrane cortex [30]. However, in the late stages of schizont maturation towards rupture, an additional source may contribute to the membrane fluctuation: (iii) the movements of mature merozoites. We observed that the E64d and EGTA-AM treated iRBCs exhibited significantly enhanced membrane fluctuations at the later time point (50–52 hpi), that were even higher than the characteristic un-infected or ring-infected RBC membrane fluctuations. This suggests the presence of an additional source of energy that drives the membrane fluctuations in rupture-arrested phenotypes, apart from those that are thermally driven.

**Figure 4:**
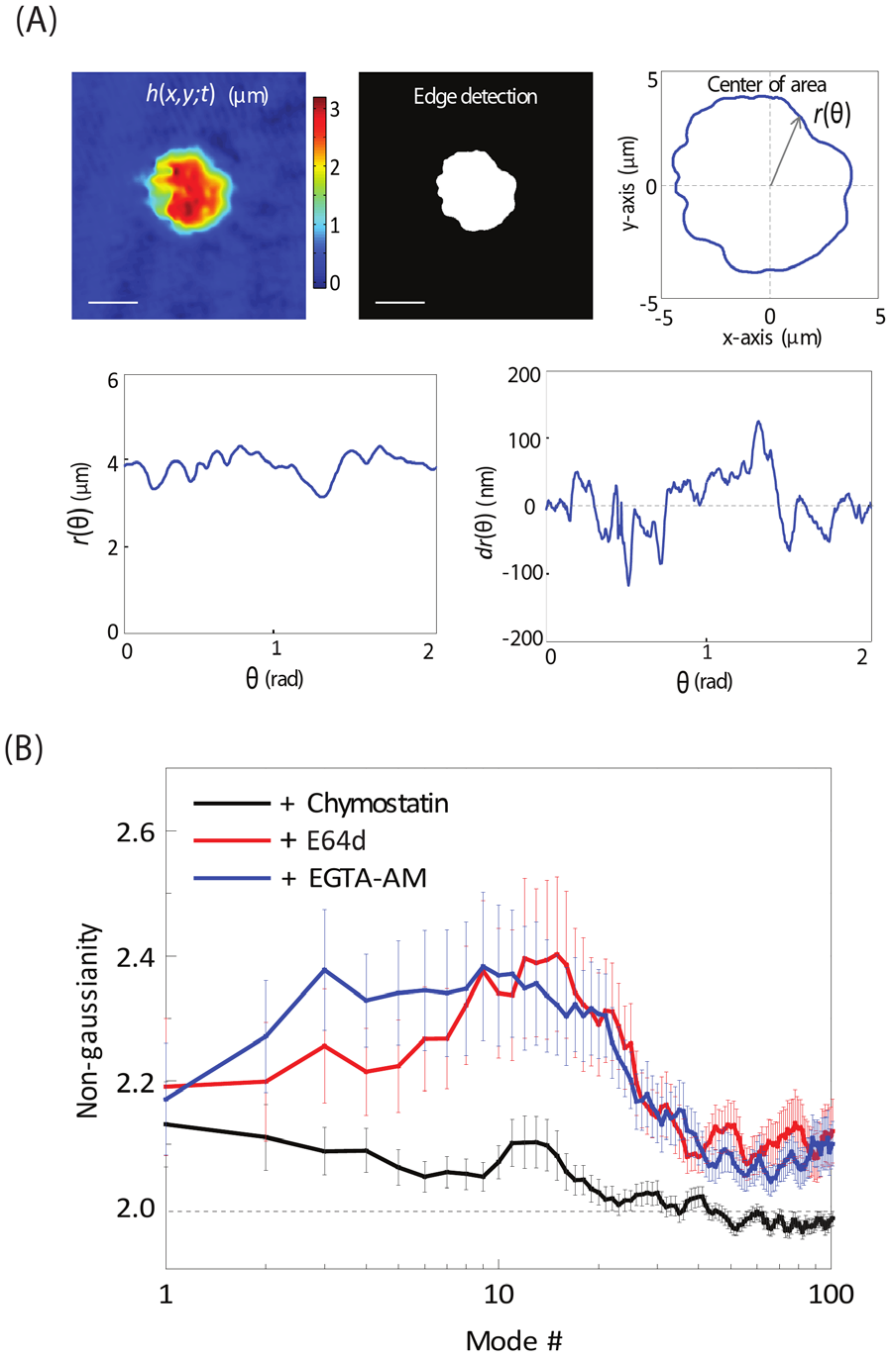
Increased membrane fluctuations in rupture-arrested iRBC membranes are likely derived from repeated merozoite impingement. (**A**) Shows procedures to analyze the membrane fluctuations of the RBC edge. The coordinates of the edge are retrieved from the measured cell thickness maps. The centre of area is calculated, and then the coordinates of the edge are converted as a function of theta by transforming into polar coordinate system. The instantaneous displacement of edge position, 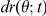, is calculated by subtracting the time-averaged edge shape, 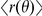, from the instant edge shape, 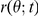. (**B**) Non-Gaussian parameter as a function of mode numbers for the iRBC treated with Chymostation (black line), E64d (red line), and EGTA (blue line), respectively. The error-bars indicate standard errors.

To elucidate increased membrane fluctuations in inhibitor-treated iRBCs, we have calculated the non-Gaussian parameters, *κ*, for the membrane fluctuations in the edges. The position of edges from the centre of iRBCs, 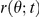, were carefully defined from the consecutive cell thickness maps, 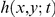, from which *κ* was calculated as a function of dynamic modes (Fig. 4A). The dynamic membrane fluctuation at the edges of infected RBCs was calculated by subtracting the averaged position of edges from the instantaneous position of edges; 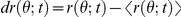, where the bracket represents the temporal average. The values for *κ* of the dynamic membrane fluctuation in the cell edge were calculated from the 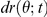 (see methods section for details).

Theoretically, *κ* = 2 for purely thermally driven Gaussian dynamics and *κ* increased above two in the presence of active non-Gaussian dynamics [31]. The averaged values of *κ* are 2.3 and 2.5 (for mode number <20), respectively for the rupture-arrested iRBCs that were treated with E64d and EGTA (Fig. 4B). For the iRBCs treated with chymostatin, however, averaged value of *κ* is 2.03. Such a significant non-Guassian response in fluctuations recorded from E64d and EGTA treated rupture-arrested iRBCs may come from two possible sources: (i) the metabolic remodeling of the membrane powered by ATP and (ii) the repeated hitting of free merozoites released upon PV breakdown on iRBC membranes. Previously, it has been shown that the effect of ATP is minimal and confined to the edges of RBC, which rules out the effect of ATP [30], [31], [32]. E64d and EGTA-treated rupture-phenotypes showed enhanced non-Gaussianity especially for mode numbers around 20, which also suggests that the colliding objects have an average size close to 1 µm (Please see the methods for the detailed explanation). Although the driving forces for merozoites to move inside the host cells upon PV break-down are not fully understood, the colliding motions of the merozoites to the host cell membrane still may exhibit non-Gaussian dynamics. This is because there are a multiple merozoites in an infected RBC and the frequency of the hitting must be limited to certain range (e.g. from a few to tens of times a second), which can result in non-thermal or non-Gaussian membrane fluctuating dynamics. Therefore, our results indicate that the enhanced fluctuations in the E64d and EGTA treated iRBCs were most likely caused by the repeated impingements of freely moving merozoites (1–1.2 µm in size). Fluctuations from chymostatin treated phenotypes followed Gaussian dynamics (i.e. *k*∼2) since merozoites were not free in the RBC cytosol, limited within the PV membrane to exert any direct hitting on the iRBC membrane.

### Optical trapping experiments show increased stiffness of rupture-arrested iRBCs

To evaluate the changes in the deformability of the rupture-impaired iRBCs, we adopted the method of optical trapping of RBCs similar to those described earlier [33], [34]. In this method, optical tweezers were used to stretch *P. falciparum* infected RBCs at various stages of parasite maturation. As RBC deformability is dependent on membrane stiffness, we used optical tweezers to obtain a quantitative measure of the membrane stiffness by determining the shear modulus *(μ)* of the RBC membrane using previously adopted methods [33], [34].

**Figure 5:**
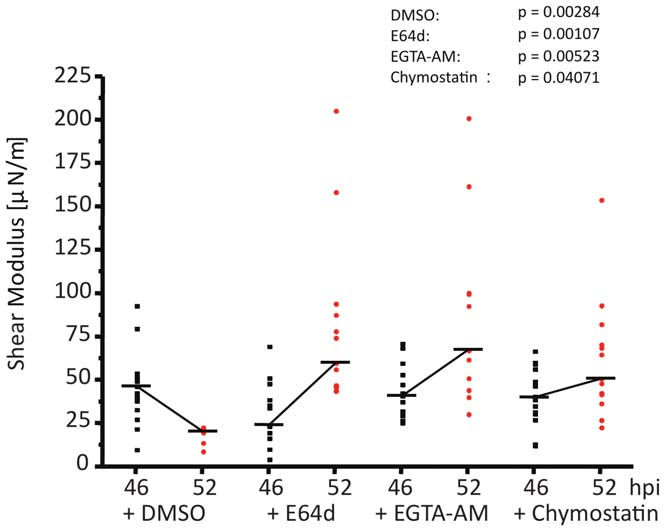
Optical trapping characterization of iRBC membrane rigidity. Results of Optical tweezers (OT) experiments with iRBCs harvested at 46 hpi and 50 hpi after DMSO, E64d, EGTA-AM and Chymostatin treatments showing shear modulus values *(μ)* were calculated for each treatment.

Optical tweezers were used to stretch iRBCs (which were treated at 44 hpi with DMSO, E64d, EGTA-AM or chymostatin), around 46 hpi (early stage) and 50–52 hpi (late stage). It was observed that the inhibitor-treated iRBCs at the early time points were susceptible to stretching slightly more as compared to the treated iRBCs at the late time points. This observation was also consistent with the calculated stiffness of the iRBCs (Fig. 5). In DMSO treated iRBCs, the shear modulus was found to decrease from *μ* = 46.99 µN/m to *μ* = 20.71 µN/m, consistent with a transition of a schizont to a ring stage parasite in the subsequent cycle. E64d-treated iRBCs showed an increase in shear modulus when going from 46 hpi (median *μ* = 24.59 µN/m) to 50–52 hpi (*μ* = 60.46 µN/m). Similarly, EGTA-AM and Chymostatin treated iRBCs showed an increase in shear modulus from *μ* = 41.35 µN/m to *μ* = 65.03 µN/m, and *μ* = 40.47 µN/m to *μ* = 51.34 µN/m respectively. Taken together, these results indicate that blocking merozoite egress in general, and specifically using protease inhibitors that permit PV membrane rupture, results in significantly increased membrane stiffness of the iRBCs. Such alterations to biophysical properties are expected to play a critical role in modulating parasite egress.

## Discussion

Parasite-mediated host cell modifications have been extensively studied in connection to infectious disease, especially in the context of malarial parasite infection of human RBCs. In this study, by coupling biochemical approaches with state-of-the-art biophysical tools of cell mechano-biology, we investigated the properties of late stage iRBCs packed with non-motile merozoites and on the ensuing mechanisms as the parasite attempts to escape the host cells, in a protease mediated pathway. Recent studies demonstrate that initial swelling of infected erythrocytes cause ‘osmotic release’ of only 1–2 merozoites, followed by ‘elastic release’ of the remaining ones characterized by rapid iRBC membrane deformations driven by the parasites [35], most likely their proteases that can modify the spectrin-based cytoskeleton. We therefore blocked parasite egress using inhibitors such as E64d, EGTA-AM and chymostatin that are known to target proteases involved in the rupture pathway, to establish a link between chemical and physical factors contributing to parasite egress.

First, we examined the morphology of iRBCs treated with inhibitors - E64d, EGTA-AM and chymostatin, all of which showed remarkable phenotypes from Giemsa stained smears, as expected. Merozoites from inhibitor treated iRBCs were unable to break open their host RBCs as late as 52 hpi forming membrane-enclosed structures that were still metabolically viable. It is known that progression of malarial parasites within human red cells results in a gradual increase in membrane stiffness [25] and increased fragility of the infected RBC as a whole. The rupture-arrested phenotypes observed from Giemsa-stained smears appeared extremely fragile under a microscope. It was further re-confirmed that E64d and EGTA supported PV membrane rupture [23] whereas chymostatin treatment prevented the rupture of either membranes. Importantly, the rupture phenotypes resulting from all these treatments demonstrated an un-altered cytoskeleton when analyzed by AFM and these cells remained intact in suspension allowing further analyses. These results indicate that cytoskeletal destabilization which is required for efficient parasite egress is blocked by different inhibitors used in this study.

We used TPM to examine the 3D-RI distribution within the iRBCs after inhibitor treatments. Typically, a schizont- stage iRBC contains a low RI area corresponding to the PV compartment and surrounding high intensity zones that represent iRBC cytosol. Schizont-stage iRBCs treated with the inhibitors, but harvested before the actual rupture time point, showed classical distribution of RI with an intact PV compartment in all treatments. However, E64d and EGTA-AM treated rupture-arrested iRBCs (∼52 hpi) demonstrated homogeneous distribution of 3D-RI. Furthermore, un-ruptured iRBCs from chymostatin treatment remained the same with no apparent change in relative distribution of RI. These results imply that the PV membrane rupture was unaffected in E64d and EGTA treatments while chymostatin blocked rupture of both the PV and iRBC membranes.

Morphology and dynamic membrane fluctuations in the *P. falciparum*-infected RBCs after inhibitor treatments were analyzed using DPM. As expected, no significant difference in morphology was noticed in inhibitor treated iRBCs that were harvested before the rupture time point (∼46 hpi). The rupture-arrested phenotypes (∼52 hpi) caused by inhibitor treatments (E64d and EGTA-AM) appeared to be unique flattened structures that were significantly different from classical spherical schizonts. Interestingly, they showed elevated membrane fluctuations around the edges of iRBCs with magnitudes even higher than those normally observed from non-infected or ring-infected RBCs. This is consistent with the merozoites released from the PV attempting to rupture its host cell by repeatedly hitting on the membrane. In contrast, chymostatin treatment showed increased membrane fluctuations within the PV compartment, suggesting ‘primed mature merozoites’ unable to escape the PV, due to a protease inactivation thereby blocking PV rupture. These observations collectively indicate that late-stages of parasite development are not affected by inhibitor treatments.

We further examined different mechanistic contributions to increased membrane fluctuations or flickering in rupture-arrested iRBCs. Apart from thermally driven fluctuations which have been extensively studied in *P. falciparum*-infected RBCs [25], [30], minor fluctuations caused by metabolic status of the cell as well as those caused by random particle movement could contribute to total membrane fluctuations. However, ATP driven fluctuations are typically not observed at the edges [30], [32] and therefore, irregular movement of free merozoites could account for the observed elevated fluctuations. In order to address this question, we analyzed non-Gaussian parameter, *κ*, *for* iRBC membrane fluctuations for rupture-arrested time points at different modes. Any fluctuation caused by random particle (merozoite) movements deviates from classical thermal Gaussian motion of the membrane. We observed that E64d and EGTA treatments generated membrane fluctuations that hugely deviated from thermal Guassian motion (*κ*∼2), while chymostatin treatment followed non-equilibrium dynamics (*κ*>2), supporting the notion that increased fluctuations were most likely caused by merozoite movements and repeated impingements on the iRBC membrane.

In order to establish the link between increased membrane fluctuations, PV disintegration and deformability changes of the inhibitor-treated iRBCs, we determined membrane stiffness in a systematic and quantitative manner for the inhibitor-treated iRBCs before and after rupture time point using the optical tweezers method. Rupture-arrest caused by E64d and EGTA-AM showed significantly increased iRBC stiffness (by factors of 2.5 and 1.6 respectively) compared to the corresponding controls analyzed before rupture time. Rupture-phenotypes from chymostatin treatment showed a 1.25 times increase in membrane stiffness compared to the corresponding earlier time point. It is known that food vacuole is the calcium rich compartment in the trophozoite stage [15], a majority of which relocalizes to the PV just prior to rupture [14] to possibly activate host calpains to facilitate rupture. Hence breakdown of PV membrane in E64d and EGTA phenotypes releases intracellular Ca^2+^ in to iRBC cytosol, that can activate calcium-dependent proteases in the red cell cytosol and trigger extensive membrane deformations reported by Abkarian et al [35], to further release merozoites. Interestingly, extra Ca^2+^ binding (in micromolar concentrations) to the RBC cytoskeleton is reported to increase the RBC rigidity [36]. EGTA-AM will have a relatively minor influence on the stiffening process, since it irreversibly chelates a significant fraction of Ca^2+^ liberated through PV rupture. Chymostatin showed little change since there was no PV breakdown to release Ca^2+^ into the iRBC cytosol. The increased membrane rigidity of the rupture-arrested phenotypes, could be also partially due to minor non-specific binding of proteins released from the PV to the iRBC cytoskeleton, altered permeability of the iRBC membrane or a combination of factors mentioned herein.

In summary, our experiments develop a comprehensive, previously unavailable, body of information on the combined effects of biochemical and biophysical factors on parasite egress from iRBCs. Our experiments offer direct evidence that during the schizont stage of parasitic life cycle, classical spherical shape of an iRBC is maintained by an integral parasitophorous vacuole. As merozoites seek to exit their host RBCs at the end of one cycle, the PV bursts first -possibly through a serine protease activity in tandem with osmotic pressure created by intake of water from the iRBC cytosol. We show that cysteine protease inhibitor- E64d and calcium chelator EGTA-AM support PV breakdown but inhibit iRBC membrane rupture generating flattened structures that had lost characteristic schizont shape. These structures show several fold-increase in membrane fluctuations that are non-Gaussian in nature. This behavior is most likely caused by random movements of primed merozoites in the iRBC cytosol, but unable to break open the cytoskeleton due to protease inactivation. However, PV breakdown renders the iRBC membrane significantly stiffer, presumably due to binding of calcium liberated from the PV to cytoskeletal components. If the merozoites are blocked from rupturing their iRBCs within a stipulated time, they become non-invasive and engender death and degradation. Hence, inhibitors that can block either serine or cysteine type proteases could potentially find their application as a potential anti-malarial strategy. Further work could also explore coupling this strategy to a specific delivery vehicle that can target malaria-infected RBCs in the blood stream.

## Materials and Methods

### Parasite culture and maintenance

Wild-type lineages of *P. falciparum* (3D7) were cultured in human red cells obtained from anonymous donors who signed a written consent. The blood collection scheme was approved by the Institutional Review Board (IRB) of National University of Singapore (NUS). Parasites were synchronized by either sorbitol selection of rings or using a magnetic separation device (Miltenyi Biotec). Cells were smeared on glass slides, fixed with methanol and stained with Giemsa for routine analyses and examined under a compound microscope (Olympus).

### Phenotypic evaluation of inhibitor treated parasites

Schizont-infected RBCs (∼44 hpi) purified on a magnetic column was incubated with 5 µM E64d (Sigma-Aldrich), 5 mM EGTA-AM (Invitrogen), or 5 µM chymostatin (Sigma-Aldrich). Controls were kept for each experiment by treating the iRBCs with DMSO. Aliquots from each treatment were harvested before the rupture time point (∼46 hpi) for analyses. DMSO-treated iRBCs were followed at regular intervals by Giemsa staining to monitor rupture. When a majority of DMSO-treated iRBCs was found ruptured (∼50 hpi), the corresponding inhibitor-treated iRBCs were harvested and these rupture-arrested phenotypes were immediately used for analyses.

### Parasite viability assay after inhibitor treatment

Parasite viability after protease inhibitor treatments was confirmed using JC-1 (5, 5′, 6, 6′-tetrachloro-1, 1′, 3, 3′-tetraethylbenzimidazol-carbocyanine iodide) staining method that distinguishes healthy and dead parasites based on mitochondrial membrane permeability [20], [21]. Mitochondrial membranes from healthy cells maintain high membrane potential and therefore staining with JC-1 gives orange-red fluorescence (590 nm) while dead cells emit green fluorescence (527 nm) upon excitation at 490 nm. To evaluate the viability of parasites, iRBCs treated with inhibitors and harvested before (46 hpi) and after the rupture time points (∼50 hpi) were stained with final concentrations of 6 µM JC-1 (Molecular Probes, Eugene, OR, USA) and 0.2 µg/ml Hoechst (Sigma-Aldrich) for 30 min at room temperature as reported before [22]. Positive controls such as treatment with Chloroquine (Sigma-Aldrich) and carbonyl cyanide 3-chlorophenylhydrazone (CCCP) (Invitrogen) were also included in the analyses. Further, the iRBCs were washed two times with PBS and analyzed on a LSRII flow cytometry instrument (BD Biosciences), with the gating parameters specifically set to look at viable schizont-stage parasites based on DNA content, JC-1 staining and size.

### AFM Imaging of iRBC surface and cytoskeletal structures

10 µl pelleted iRBCs after inhibitor treatments was allowed to bind to poly-l-lysine (Sigma Aldrich) pre-coated glass slide for 10 min and washed twice. Cells attached to the slides were then fixed in 1% glutaraldehyde (Electron Microscopy Science) for 30 min and washed in distilled water and dried in a vacuum desicator. Imaging was performed in tapping mode in air on a Dimension 3100 AFM system equipped with Nanoscope III controller (Veeco Instrument, CA) and processed in Nanoscope V5.31 (Veeco Instrument).

For imaging the cytoskeletal network, the sample was smeared on a clean glass slide and dried in a dry cabinet (Bossmen Inc., Taiwan, ROC). Imaging was performed in tapping mode in air on a Dimension 3100 AFM system equipped with Nanoscope III controller (Veeco Instrument, CA). Super sharp AFM probes (SSS-NCHR, Nanosensor, Neuchatel, Switzerland) were used to maximize the resolution to reveal the cytoskeleton structures. Images were processed in Nanoscope V5.31 (Veeco Instrument, Santa Barbara, CA).

### Targeting vector construct to generate preprocathepsin-GFP parasites

The pARL-STEVOR full vector [37] was linearized at the BglII and AvrII sites to excise the existing STEVOR sequence. A fragment encoding the 5′-UTR together with the first exon of the preprocathepsin c gene (PFL2290w) was amplified using the PFL2290w_BglII (AGTCAGATCTGCACCATCACATTTTACAGTCC) and PFL2290w_AvrII (AGTCCCTAGGTGGGTTTAAGTTATCCAAATTTCT) primers to create the pARL-PPC targeting vector. Parasites were transfected with 100 µg plasmid DNA and drug resistant transfectants selected with 5 nm WR99210 (Jacobus Pharmaceauticals, NJ, USA).

### Tomographic phase microscopy

Tomographic phase microscopy (TPM) was employed to measure the 3-D distribution of refractive index [25], [26] within inhibitor-treated iRBCs before and after rupture time-points. In TPM, the sample-induced optical phase shift at various illumination angles are measured using a phase-shifting heterodyne interferometer. The angle of illumination ranges from −60° to 60° and angular step is 0.2°. The entire angular range was scanned within 5 s. With the set of angular projection phase images, a back-projection algorithm is used to calculate a 3D reconstruction of the sample RI. The custom built microscope is equipped with a 100× objective lens (oil immersion, 1.4 NA). The CMOS camera (FASTCAM 1024 PCI, Photron Inc., San Diego, CA) was used to record the interferometry. The transverse and axial resolutions are 0.3 µm and 0.6 µm, respectively, and the accuracy of RI measurement is 0.001.

### Diffraction phase microscopy

To quantitatively measure the cell thickness map and its dynamics from inhibitor-treated iRBCs, we used Diffraction Phase Microscopy (DPM), which employs the principle of laser interferometry in a common path geometry [27], [28]. It provides full-field quantitative phase images of RBCs with high optical path-length stability. An Ar^2+^ laser (λ = 514 nm) was the source of illumination for an inverted microscope (IX71, Olympus). The microscope was equipped with a 40× objective lens (0.75 NA), which facilitates a diffraction-limited transverse resolution of ∼400 nm. With the additional optics used outside the microscope, the overall magnification of the system was approximately 200×. EMCCD (Photonmax 512B, Princeton Instruments Inc., Trenton, NJ) was used to image interferogram. The DPM measured the electric field at the image plane, 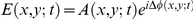, where *A(x,y;t)* and *Δφ(x,y;t)* are amplitude and phase delay map of the field at the location *(x,y)* and time *t*, respectively. The instantaneous cell thickness map can be retrieved from the phase delay map as 
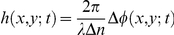
. The refractive index contrast *Δn* between the RBC and the surrounding medium mainly originated from erythrocyte hemoglobin, which is optically homogeneous in the cytosol. We used the *Δn* of red blood cells calibrated from TPM. The DPM optical path-length stability is 2.4 mrad, which corresponds to a membrane displacement of 3.3 nm in the RBC membrane fluctuation [27].

### Non-Gaussian parameter

The non-Gaussian parameter, *κ*, is defined by the second and fourth moments of the dynamic membrane displacement. First, the dynamic membrane fluctuations in the cell edge the *dr(θ;t)*, were decomposed into Fourier modes *dr(q, Δt)*, using two consecutive Fourier transforms; lag time is *Δt*, and the spatial frequencies *q = 2π/Λ*, where *Λ* is a spatial distance. Since the mode number, *#*, is directly related to the spatial frequency; # = q/(π^*^circumference), the dynamic fluctuation in the cell edge can be represented in terms of mode number and lag time; *dr(#, Δt)*. The detailed dynamic analysis based on mode number can be found elsewhere [32]. *κ* was then calculated from the second and forth moments of the membrane height displacement as, 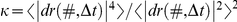.

The mode number, *#* is directly related to the wavelength of the fluctuation; # = 2 * circumference / *Λ*. For example, the mode number of 20 corresponds to the fluctuation *Λ* of 2 * circumference / 20. Considering the average diameter of RBC is 8.5 µm (which means the average circumference is 26 µm), *Λ* of the fluctuation corresponding to the mode number of 20 is 2.6 µm. In order to generate the enhanced fluctuation with the wavelength of 2.6 µm, the size of colliding particle should be the half of the wavelength, which is 1.3 µm.

### Deformability measurements of iRBC membrane using optical tweezers

Streptavidin-coated polystyrene beads of 3 µm diameter (Micromer-M, Micromod) were incubated for 40 min at 4°C in 1 mg/ml Con A (Sigma-Aldrich). Beads were further washed three times with 0.1 mg/ml BSA-PBS and collected by centrifuging at 16,000× g and stored in 0.1 mg/ml BSA-PBS at 4°C until used.

The force-displacement response of a malaria-infected RBC undergoing stretch was measured using an optical tweezers system (OT) comprising of an inverted microscope (TE300, Nikon), optics and a 2 W infrared laser (1064 nm). The iRBCs pre-treated with protease inhibitors was mixed with a streptavidin- Con A beads previously prepared and loaded into a liquid chamber comprising a thin piece of parafilm sandwiched between two glass coverslips. Individual iRBCs with one side attached to the cover slip at the bottom and the diametrically opposite side attached to a single bead were precisely located. Increasing stretching forces were applied to the selected iRBCs step-wise and the images recorded during the stretching procedure. Membrane stiffness information as given by shear modulus values *(μ)* was computed by analyzing the images and fitting the experimental data to analytical expressions derived from computational modeling as described previously.

### Statistical Analysis

Reported shear modulus values for optical tweezers experiments are median values from the multiple measurements shown in Fig. 4. P values are calculated by two-tailed two sample T- test between various test conditions.

## Supporting Information

Figure S1 **Profiling of parasite viability after inhibitor treatment.** (**A**) Late-stage (∼44 hpi) parasite infected RBCs were treated with (1) 50 nM carbonyl cyanide 3-chlorophenylhydrazone (CCCP) or (2) 25 µM Chloroquine each or 2 hours. Samples were further stained with JC-1 and Hoechst to evaluate induction of cell death. Flow cytometric analyses demonstrated significant death of parasites as indicated by loss of mitochondrial membrane potential within 2 hours. (**B**) Original flow plots corresponding to Fig. S1A highlighting transition of healthy parasites to dead ones upon the following treatments; 50 nM carbonyl cyanide 3-chlorophenylhydrazone (CCCP) or 25 µM Chloroquine (**C**) DMSO, E64d, EGTA & Chymostatin treated iRBcs were harvested before (∼46 hpi) and after rupture time point (∼50 hpi) stained with JC-1 and Hoechst. The major fraction of the parasites remained healthy as represented in the flow plot after 6 hours of incubation with the inhibitors.
(PDF)

Figure S2 **Re-distribution of merozoites inside iRBCs treated with protease inhibitors.** *P. falciparum* PC-GFP line expressing cytosolic GFP was treated with DMSO, E64d, EGTA-AM and Chymostatin at 44 hpi and harvested at 46 hpi and 50–52 hpi and lysed with 5% sorbitol. Lysates from this experiment was resolved by SDS-PAGE, transferred to nitrocellulose membrane and probed for GFP. At 46 hpi, no GFP signal was observed in any of the lysates while by 52^nd^ hour, E64d and EGTA-treated samples showed enhanced signals for GFP. This was not observed in chymostatin treated iRBCs at 52 hpi. A luminal RBC protein- calpain- served as the loading control.
(PDF)

Figure S3 **AFM imaging of rupture-arrested iRBCs.** iRBCs treated with protease inhibitors were harvested at 46 hpi and 52 hpi and were examined. Images of outer surfaces from the rupture-arrested iRBCs (Upper Panel) and cytoskeletal structures (Lower Panel) were captured using an AFM device.
(PDF)
