# Supplementary figures and images for: Biophysics of Malarial Parasite Exit from Infected Erythrocytes
Source: PLoS One. 2011 Jun 17;6(6):e20869. doi: 10.1371/journal.pone.0020869 (PMC3117855; doi:10.1371/journal.pone.0020869)

Figure. S1

A

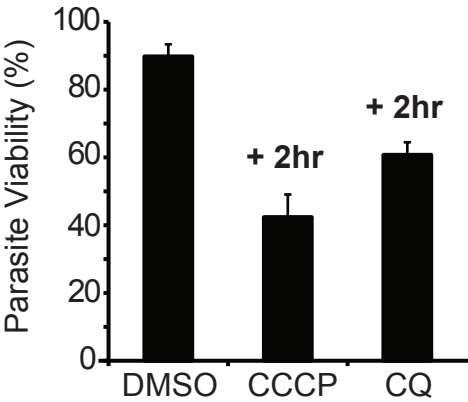

B

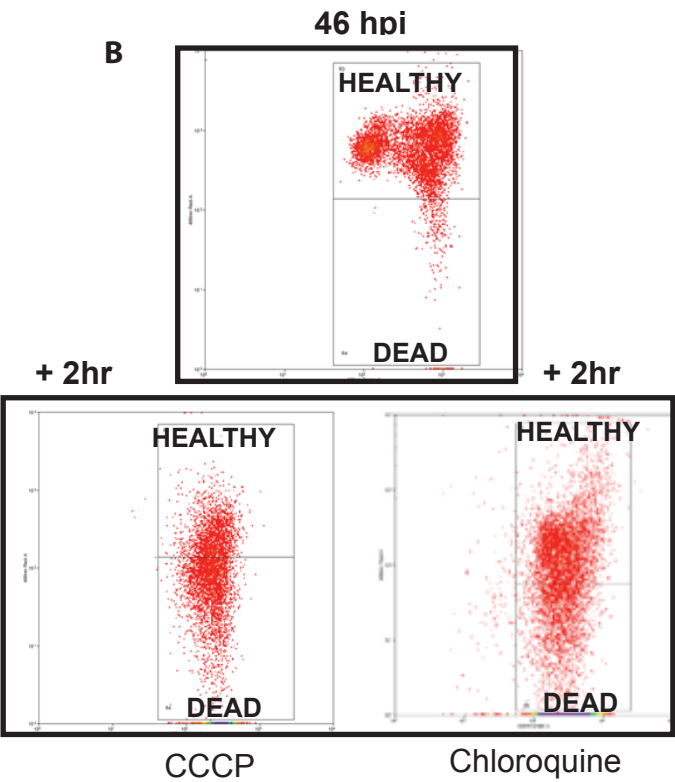

C

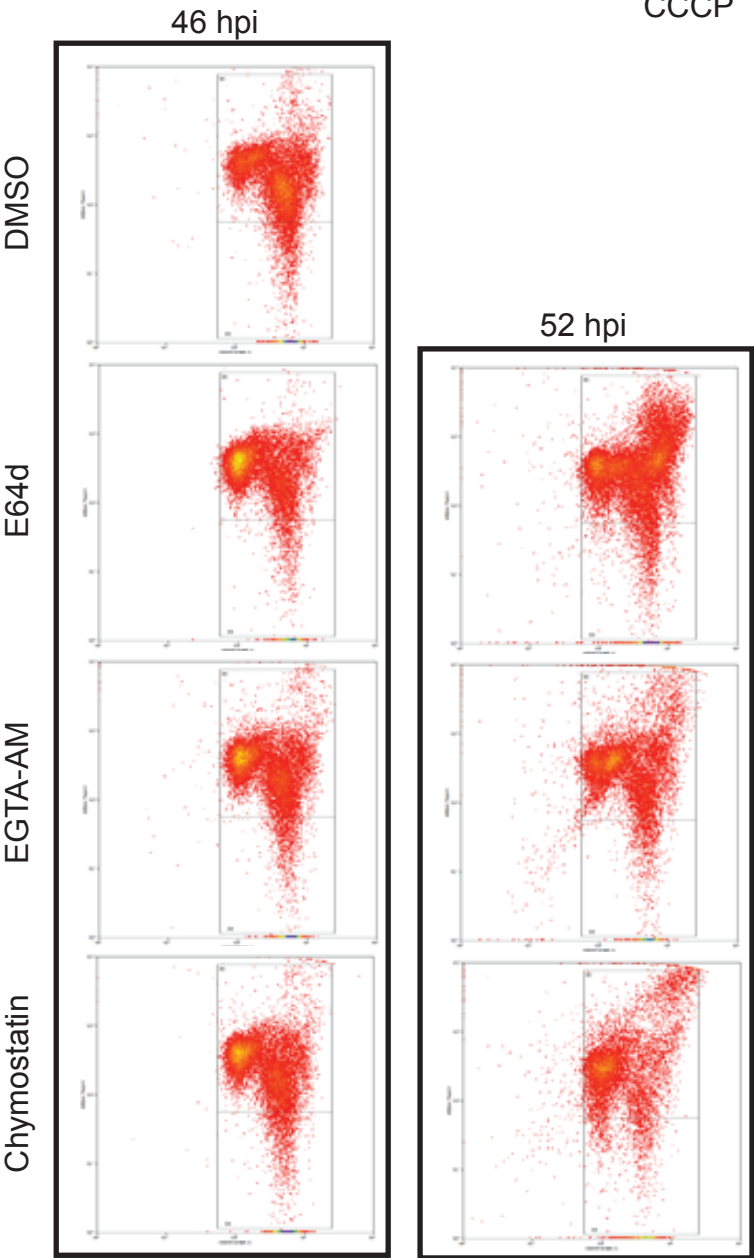

Supplement: Figure S1 — Profiling of parasite viability after inhibitor treatment. (A) Late-stage (∼44 hpi) parasite infected RBCs were treated with (1) 50 nM carbonyl cyanide 3-chlorophenylhydrazone (CCCP) or (2) 25 µM Chloroquine each or 2 hours. Samples were further stained with JC-1 and Hoechst to evaluate induction of cell death. Flow cytometric analyses demonstrated significant death of parasites as indicated by loss of mitochondrial membrane potential within 2 hours. (B) Original flow plots corresponding to Fig. S1A highlighting transition of healthy parasites to dead ones upon the following treatments; 50 nM carbonyl cyanide 3-chlorophenylhydrazone (CCCP) or 25 µM Chloroquine (C) DMSO, E64d, EGTA & Chymostatin treated iRBcs were harvested before (∼46 hpi) and after rupture time point (∼50 hpi) stained with JC-1 and Hoechst. The major fraction of the parasites remained healthy as represented in the flow plot after 6 hours of incubation with the inhibitors. (PDF) [file pone.0020869.s001.pdf]

Figure. S2

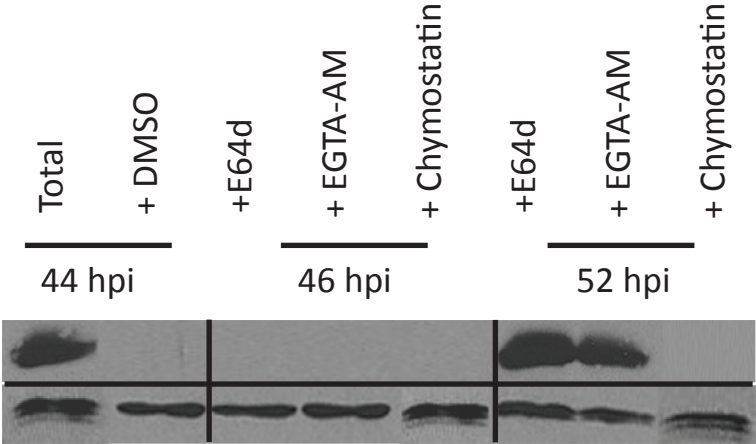

Supplement: Figure S2 — Re-distribution of merozoites inside iRBCs treated with protease inhibitors. P. falciparum PC-GFP line expressing cytosolic GFP was treated with DMSO, E64d, EGTA-AM and Chymostatin at 44 hpi and harvested at 46 hpi and 50–52 hpi and lysed with 5% sorbitol. Lysates from this experiment was resolved by SDS-PAGE, transferred to nitrocellulose membrane and probed for GFP. At 46 hpi, no GFP signal was observed in any of the lysates while by 52nd hour, E64d and EGTA-treated samples showed enhanced signals for GFP. This was not observed in chymostatin treated iRBCs at 52 hpi. A luminal RBC protein- calpain- served as the loading control. (PDF) [file pone.0020869.s002.pdf]

Figure. S3

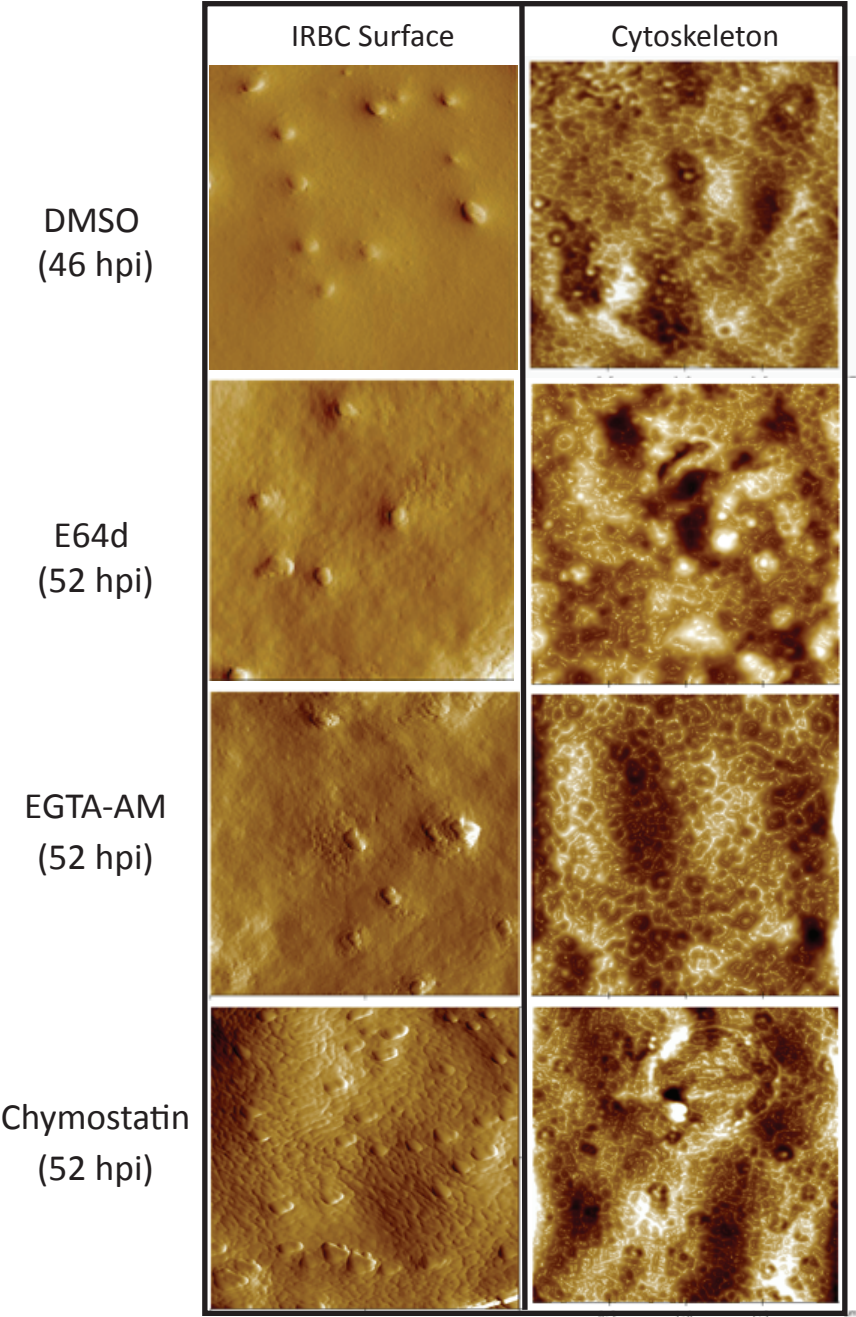

Supplement: Figure S3 — AFM imaging of rupture-arrested iRBCs. iRBCs treated with protease inhibitors were harvested at 46 hpi and 52 hpi and were examined. Images of outer surfaces from the rupture-arrested iRBCs (Upper Panel) and cytoskeletal structures (Lower Panel) were captured using an AFM device. (PDF) [file pone.0020869.s003.pdf]
